# Discovery of Enterovirus A71-like nonstructural genomes in recent circulating viruses of the *Enterovirus A* species

**DOI:** 10.1038/s41426-018-0107-0

**Published:** 2018-06-21

**Authors:** Kuo-Ming Lee, Yu-Nong Gong, Tzu-Hsuan Hsieh, Andrew Woodman, Nynke H. Dekker, Craig E. Cameron, Shin-Ru Shih

**Affiliations:** 1 Research Center for Emerging Viral Infections, College of Medicine, Chang Gung University, Taoyuan, Taiwan; 2 Department of Medical Biotechnology and Laboratory Science, College of Medicine, Chang Gung University, Taoyuan, Taiwan; 3 Department of Biochemistry and Molecular Biology, The Pennsylvania State University, University Park, PA 16802 USA; 4 Department of Bionanoscience, Kavli Institute of Nanoscience, Delft University of Technology, Van der Maasweg 9, Delft, 2629 HZ The Netherlands; 5 Department of Laboratory Medicine, Linkou Chang Gung Memorial Hospital, Taoyuan, Taiwan; 6 Research Center for Chinese Herbal Medicine, Research Center for Food and Cosmetic Safety, and Graduate Institute of Health Industry Technology, College of Human Ecology, Chang Gung University of Science and Technology, Taoyuan, Taiwan

## Abstract

Enterovirus A71 (EV-A71) is an important nonpolio enterovirus that causes severe neurological complications. In 1998, Taiwan experienced an EV-A71 outbreak that caused 78 deaths. Since then, periodic epidemics of EV-A71 associated with newly emerging strains have occurred. Several of these strains are known to be recombinant; however, how these strains arose within such a short period of time remains unknown. Here, we sequenced 64 full-length genomes from clinical isolates collected from 2005 to 2016 and incorporated all 91 Taiwanese genomes downloaded from the Virus Pathogen Resource to extensively analyze EV-A71 recombination in Taiwan. We found that the B3 subgenotype was a potential recombinant parent of the EV-A71 C2-like and C4 strains by intratypic recombination. Such B3-similar regions were also found in many cocirculating coxsackieviruses belonging to *Enterovirus A* species (EV-A) through a series of intertypic recombinations. Therefore, locally enriched outbreaks of cocirculating viruses from different genotypes/serotypes may facilitate recombination. Most recombination breakpoints we found had nonrandom distributions and were located within the region spanning from the boundary of P1 (structural gene) and P2 (nonstructural) to the *cis*-acting replication element at P2, indicating that specific genome reassembly of structural and nonstructural genes may be subject to natural selection. Through intensive recombination, 11 EV-A71-like signatures (including one in 3A, two in 3C, and eight in 3D) were found to be present in a variety of recently cocirculating EV-A viruses worldwide, suggesting that these viruses may be targets for wide-spectrum antiviral development.

## Introduction

Enterovirus A71 (EV-A71), a member of the nonpolio enterovirus family, belongs to the *Enterovirus A* species (EV-A) of Picornaviridae^1^. Notably, infection with EV-A71 in children under 5 years of age sometimes leads to severe neurological complications (e.g., brainstem encephalitis, meningitis, and acute flaccid paralysis) and even death owing to cardiopulmonary failure^1–3^. EV-A71 is a nonenveloped small RNA virus with a positive, single-stranded RNA genome^1^. The viral genome can be directly translated into a polyprotein consisting of the structural (P1) and nonstructural (P2 and P3) regions; this polyprotein is subjected to a series of proteolytic cleavages to generate functional proteins, including structural proteins (VP1–4) functioning in capsid assembly and nonstructural proteins (2A–C, 3A–D) required for viral replication^1^. Based on the nucleotide sequence of the VP1 protein, EV-A71 is classified into seven genotypes (A–G)^4–6^. Genotypes B and C can be further divided into subgenotypes from B0 to B5 and from C1 to C5, respectively^7^. Outbreaks caused by these variable genotypes have been reported^8^.

In 1969 and the early 1970s, EV-A71 genotype A caused outbreaks in the United States of America (USA). However, genotype A did not recur until 2008, and the intervening worldwide epidemics were found to be caused by other genotypes^8^. From the 1970s to the late 1980s, genotype B dominated and led to outbreaks in the USA, Japan, Australia, and Europe. In the late 1980s, the prevalent genotype changed to genotype C, which is currently active outside the Asia-Pacific region. However, after the 1975 Bulgaria and 1978 Hungary outbreaks, no severe outbreaks occurred until 1997^7,8^; since then, several EV-A71 outbreaks have accompanied fatal hand–foot–mouth disease (HFMD) cases in various countries in the Western Pacific region, and the threat continues^8^. Thus, EV-A71 has been selected by the World Health Organization as one of the top five viruses in the post-polio eradication era^9^. These recent outbreaks were associated with newly emerging strains, including the recombinant B3, B4, C2, and C4 subgenotypes^7,8^. Notably, recombination is believed to play a more important role than that of mutations in the evolution of EV-A71^7,10^. Although clinical trials of the EV-A71 vaccine are currently ongoing^11^, novel vaccine-resistant strains might appear as a result of recombination. Therefore, characterization of the mechanisms of viral recombination remains essential.

In Taiwan, EV-A71 has become a long-term pathogen and can be traced back to as early as 1980^3^; however, the first severe outbreak occurred in 1998, and Taiwan experienced the most severe EV-A71 outbreak on record. In a subsequent outbreak in 2000–2002, 846 severe cases and 129 deaths were reported^2,3^. Thus, enterovirus infection has been evaluated as an important infectious disease in Taiwan. From 1989 to 2009, physicians and hospitals used sentinel surveillance systems to monitor highly infectious diseases; now, computerized systems carry this responsibility. Additionally, a laboratory-based virological surveillance system was established in 2000 to focus on influenza virus and enterovirus infections^3^, and reporting of severe cases is now mandatory in Taiwan.

Here, we evaluated the evolution and recombination of different genotypes of EV-A71 and several cocirculating EV-A viruses in Taiwan based on full-genome sequence analyses. Our results provide insights into the crucial role of the similar nonstructural regions via a series of recombination events associated with various serotypes, which may be triggered by the emergence of the temporal EV-A71 B3 strain. These results may facilitate the development of wide-spectrum antivirals against cocirculating EV-A strains.

## Results

### Molecular epidemiological analysis of EV-A71 from 2005 to 2016 in Taiwan

**Fig. 1:**
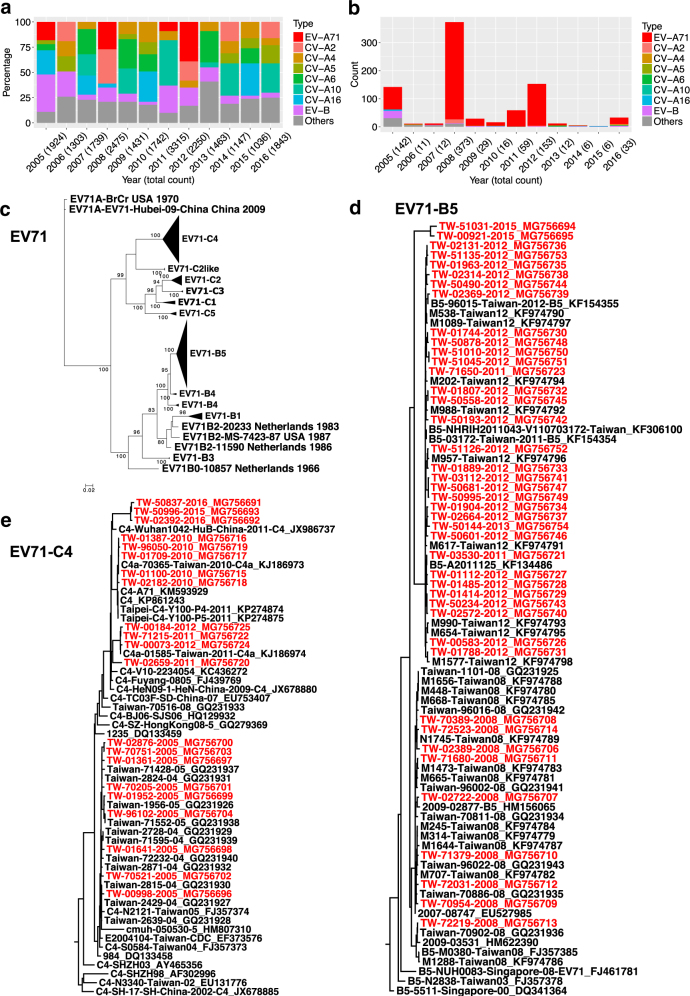
Enterovirus surveillance reports in Taiwan since 2005 and phylogenetic tree of Taiwanese EV-A71 full-genome sequences. **a** Percentages of enterovirus infections since 2005, including EV-A71 (marked in red), six CV types (A2, A4, A5, A6 A10, and A16 in different colors), EV-B (purple), and other species (gray) in Taiwan. EV-B included CV types (B1–6 and A9) and echovirus types 3, 6, 11, 18, and 30. Counts of total cases reported in each year are also shown in parentheses. **b** Counts of severe complications are further summarized. **c** Compressed ML tree of Taiwanese strains in various subgenotypes (A, B0–5, and C1–5). Significant bootstrap support values greater than 70% are indicated at major nodes. **d** B5 and **e** C4 subtrees are shown. The tip labels of the 63 strains in B5 and C4 sequenced in this study are colored in red

We summarized epidemiological reports of enterovirus infections from the Taiwan Centre for Disease Control in Fig. 1. More than 1000 enterovirus infection cases have been reported annually since 2005 in Taiwan, and both EV-A and EV-B viruses were common (Fig. 1a). No dominant serotype has been observed since 2005, and EV-A71 infections (Fig. 1a, marked in red) had a lower prevalence than those of the other cocirculating types, except in 2012. However, most severe cases were associated with EV-A71 infections (Fig. 1b), and much higher numbers of severe cases were reported in 2005, 2008, 2011, and 2012, correlated with EV-A71 outbreaks^12,13^.

**Fig. 2:**
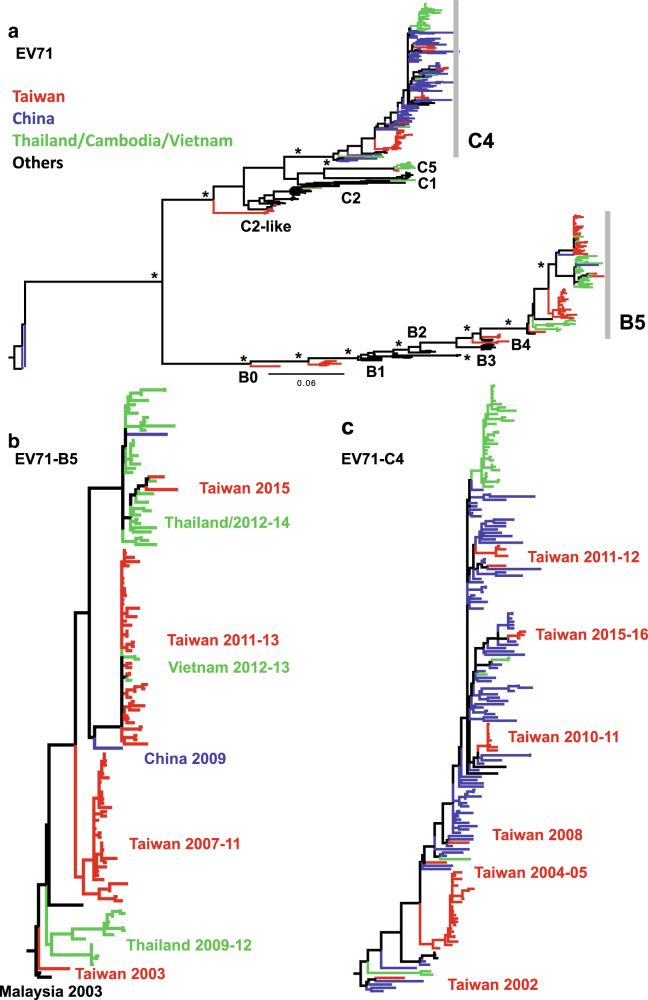
ML tree of the VP1 gene in Taiwanese and worldwide EV-A71 strains. **a** Compressed tree, including Taiwanese and worldwide strains. Significant bootstrap support values greater than 70% are indicated by asterisks at the major nodes. Strains isolated from Taiwan, China, Cambodia/Thailand/Vietnam, and other countries are marked in different colors. **b** B5 and **c** C4 subtrees are shown

Sixty-four clinical isolates collected from 2005 to 2016 were sequenced to investigate EV-A71 recombination. Yearly counts are presented in Table 1. For the purpose of recombination analysis, all of the 91 full-length genomes isolated from Taiwan were downloaded from the Virus Pathogen Resource (ViPR) and subjected to the following examinations. A maximum likelihood (ML) tree was inferred using all Taiwanese genomes and published reference sequences with known subgenotypes (EV-A71 A, B0–B5, and C1–C5; Fig. 1c). The genotype distribution of the Taiwanese strains is summarized in Table 1. Except for one C5 strain collected in 2007, all our clinical isolates belonged to the B5 and C4 subgenotypes. The B5 and C4 subtrees are shown in Fig. 1d, e, respectively. The B5 strain showed a ladder-like distribution in the phylogenetic tree (Fig. 1d). The strains collected in each year formed a distinct clade, and the consecutive replacement of the older clades with the more recent clades indicated the continuous evolution of the B5 strain after its divergence from the B4 strain. However, the most recent B5 strains seemed to represent a new branch (Fig. 1d, upper), which is further discussed in Fig. 2b. The EV-A71 C4 strains collected in different years also belonged to different clades (Fig. 1e). In contrast, these clades showed a scattered distribution, meaning that multiple lineages of the C4 strain may coexist and be transmitted over time.

**Table 1:** Taiwanese genomes of EV-A71 analyzed in this study

Year	Counts (genotype) of sequences acquired in this study	Counts (genotype) of sequences downloaded from ViPR
1986		6 (B1)
1998		6 (C2)
1999		1 (B4)
2000		1 (B4)
2001		1 (B4)
2002		1 (C4)
2003		1 (B5)
2004		11 (C4)
2005	9 (C4)	5 (C4)
2007	1 (C5)	1 (B5), 1 (C5)
2008	9 (B5)	19 (B5), 3 (C2-like), 1 (C4)
2009		2 (B5)
2010	5 (C4)	1 (C4)
2011	2 (B5), 2 (C4)	8 (B5), 6 (B4), 4 (C4)
2012	28 (B5), 2 (C4)	10 (B5), 1 (C2)
2013	1 (B5)	1 (C2)
2014		
2015	2 (B5), 1 (C4)	
2016	2 (C4)	
Total	64	91

Interestingly, a periodic switching between the EV-A71 C4 and B5 strains occurred in Taiwan during the years from 2005 to 2016 (Table 1). To elucidate whether this genotype switching may be caused by the transmission of different EV-A71 strains from other countries, the VP1 genes of genomes collected worldwide were obtained from ViPR and phylogenetically analyzed (Fig. 2a). The details of each analyzed sequence can be found in Supplementary Table 1. Since B5 and C4 were the dominant circulating EV-A71 strains in Taiwan, we examined their relationships with the same strains circulating in nearby countries, including China, Cambodia, Thailand, and Vietnam (Fig. 2b, c). In the B5 tree, most sequences came from Taiwan; indeed, Taiwan has been the only country to suffer from EV-A71 outbreaks caused by the B5 strain. Sequences from other countries collected at similar times formed clusters with Taiwanese sequences (Fig. 2b, marked in red). For example, Taiwanese strains in 2003 (TW/2003) were closely related to strains from Malaysia in the same year. TW/2011-13 were clustered together, but they also clustered with two Vietnam/2012-13 strains and one China/2009 strain. In contrast, the new B5 branch may be derived from recent sequences found in Thailand. Two TW/2015 strains were closely related to the Thailand/2012–14 strains (Fig. 2b, upper), and the older Thailand strains (before 2012) were within the other cluster containing the TW/2003 strains rather than that containing the TW/2007-11 strain (Fig. 2b, lower). Thus, the B5 strain may have first been transmitted from Taiwan to Thailand, where the virus independently evolved. Later, the locally evolved Thailand B5 strain was transferred back to Taiwan. Regarding the C4 strain, Taiwanese strains isolated after 2005 showed higher similarity to those isolated from China (Fig. 2c). Unlike that of the B5 strains, a scattered distribution of the C4 strains was observed (Fig. 2c). This result indicated that their genetic clades were not correlated with isolation year, despite the close relationships between strains from Taiwan and China in similar isolation years. Considering the different antigenicities of the B5 and C4 strains, which could alter herd immunity^13^, genotype switching in Taiwan may be related to the frequent transmission of different strains outside the Taiwan region.

### Interconnection of circulating EV-A71 by intratypic recombination

**Fig. 3:**
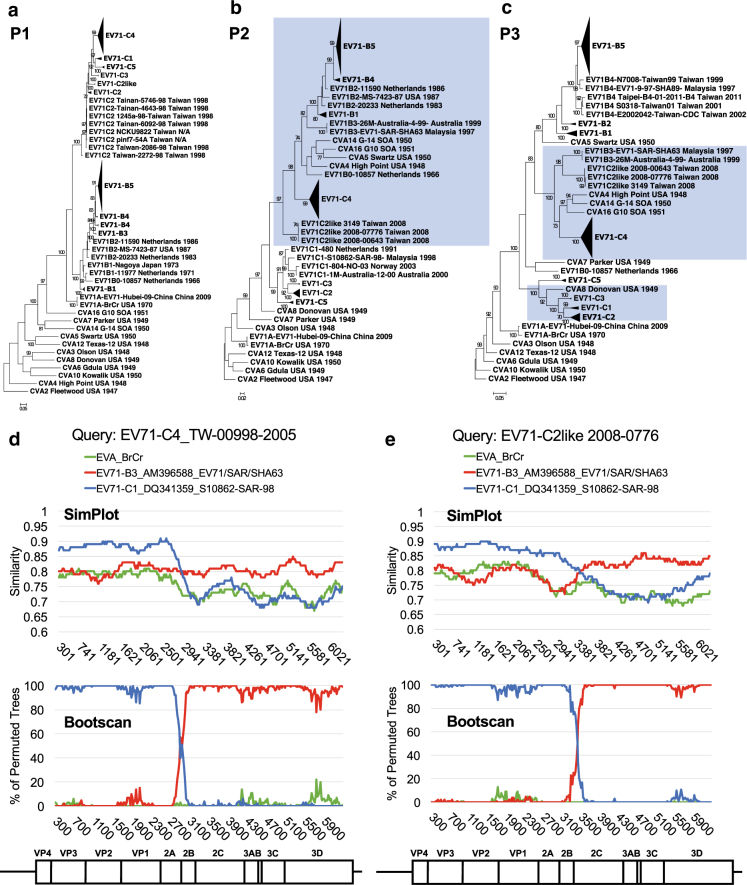
Intratypic recombination revealed by cross-genotypic patterns in ML trees of subgenomic EVA-71 sequences. Cross-genotypic patterns are found in the compressed trees of the (**a** P1, **b** P2, and **c** P3) regions, rooted by the oldest prototype strain (CVA2-Fleetwood), which was isolated in 1947. Comparing to the P1 region, two reclusterings containing EV-A71 C2-like/C4 with B3 and CV-A8 with EV-A71 C in the P2 and P3 region are highlighted in blue. Through intratypic recombination, the B3 subgenotype is a potential origin of recombinants. **d** EV-A71 C4 and **e** C2-like strains were detected by SimPlot (upper panel) and Bootscan (lower panel) analyses. Reference strains, as noted, are marked in different colors. Numbers indicate nucleotide positions in the CDS corresponding to the viral genome

Recent EV-A71 outbreaks have been characterized by an association with newly emerging subgenotypes^8^. Both the C2 and C4 strains are recombinogenic and carry partial genomes derived through intertypic recombination with coxsackievirus (CV)-A8 and CV-A16, respectively^13,14^. Furthermore, the B4 strain is an example of intratypic recombination within EV-A71 genotype B^13^. The emergence of various EV-A71 strains within a decade may be attributable to regionally enriched, large-scale outbreaks that can increase the risk of coinfection, a key requirement for recombination^15^. To examine this possibility, we evaluated Taiwanese full-genome sequences that consisted of a variety of genotypes/subgenotypes (Table 1) and explored the relationships among these newly emerging strains. EV-A71 and prototype CV (including types A2–A8, A10, A12, A14, and A16) sequences of EV-A were compared to reveal their recombinogenic properties. Breakpoints in EV-A71 recombination are usually located in the P2 and P3 regions^16^. Therefore, we first reconstructed ML phylogenetic trees of the P1, P2, and P3 regions (Fig. 3a–c, respectively). Possible recombination events were revealed by changes in the tree positions of analyzed sequences in the subgenomic phylogenies^17^.

In the phylogenetic tree constructed via the P1 region and rooted with the oldest strain (CVA2-Fleetwood), distinct clades representative of each genotype/subgenotype were observed, and all sequences of prototype CV appeared as an outgroup (Fig. 3a). In contrast, cross-genotypic patterns in the CV-A and EV-A71 sequences were identified in the P2 and P3 phylogenetic trees (Fig. 3b, c). The inconsistency of these phylogenies reflected recombination events. For example, CV-A8 was reported as the recombination parent of EV-A71 C2^13^, and this result is reflected by their coclustering in the P3 region (Fig. 3c, highlighted in blue), indicating that the high sequence similarity between CV-A8 and EV-A71 genotype C may have facilitated the recombination and emergence of the EV-A71 C2 strain. Similar reclustering also occurred in the C2-like and C4 strains. In the P2 phylogenetic tree, the C2-like strain was an outlier of genotype C of EV-A71, and the C4 cluster was closely related to genotype B and several prototype CVs (Fig. 3b). The clustering pattern changed again in the P3 phylogenetic tree, and a new cluster containing EV-A71 B3, C2-like, and C4 was formed. Additionally, prototypes CV-A4, CV-A14, and CV-A16 were redistributed into the same cluster (Fig. 3c). Given that the EV-A71 C4 strain was regarded as a double-recombinant virus containing EV-A71 genotype B-like P2 and CV-A16-like P3 regions and that the B3 strain was a recombinant with a CV-A16-like 3D region^14,18,19^, our results suggested that the EV-A71 B3 strain may be the possible recombination parent of the C4 strain. Similarly, the C2-like strain may be an uncharacterized recombinant EV-A71 that also originated from the B3 strain. These predictions were confirmed by SimPlot analysis (Fig. 3d, e). When comparing the C4 strain to reference strains including the EV-A71 genotypes A, B3, and C1, its 5′ region showed a higher similarity (approximately 88%) to that of EV-A71 genotype C1; however, the similarity decreased at the boundary of the 2A/2B coding region, and this effect was accompanied by an increased similarity (approximately 80%) to B3 toward the 3′ half of the viral genome. The recombinant breakpoint was mapped to approximately nucleotide position 2881 of the coding sequence (CDS) (Fig. 3d). The shift of predominant similarity from one reference strain to another in different genomic regions was also observed when we queried C2-like strain sequences. The C2-like genome contained genotype C- and B3-similar sequences at the 5′ and 3′ regions, respectively. However, the breakpoint of the C2-like strain was mapped to the downstream 2C coding region (approximately nucleotide 3481 of the CDS), near the structural *cis*-acting replication element (Fig. 3e). Since the EV-A71 B3 strain was also the recombination parent of the B4 strain^13^, our data suggested that a temporally circulating EV-A71 strain B3 may serve as an important intermediate leading to the emergence of diverse EV-A71 strains. Although we attempted to remove sample size limitations in the interpretation of the phylogenetic trees (by downloading all of the full-length genomes isolated in Taiwan for the current study), some differences may still exist between the published full-length genomes and the actual viral population.

### Extensive genomic recombinations among the cocirculating enteroviruses

**Fig. 4:**
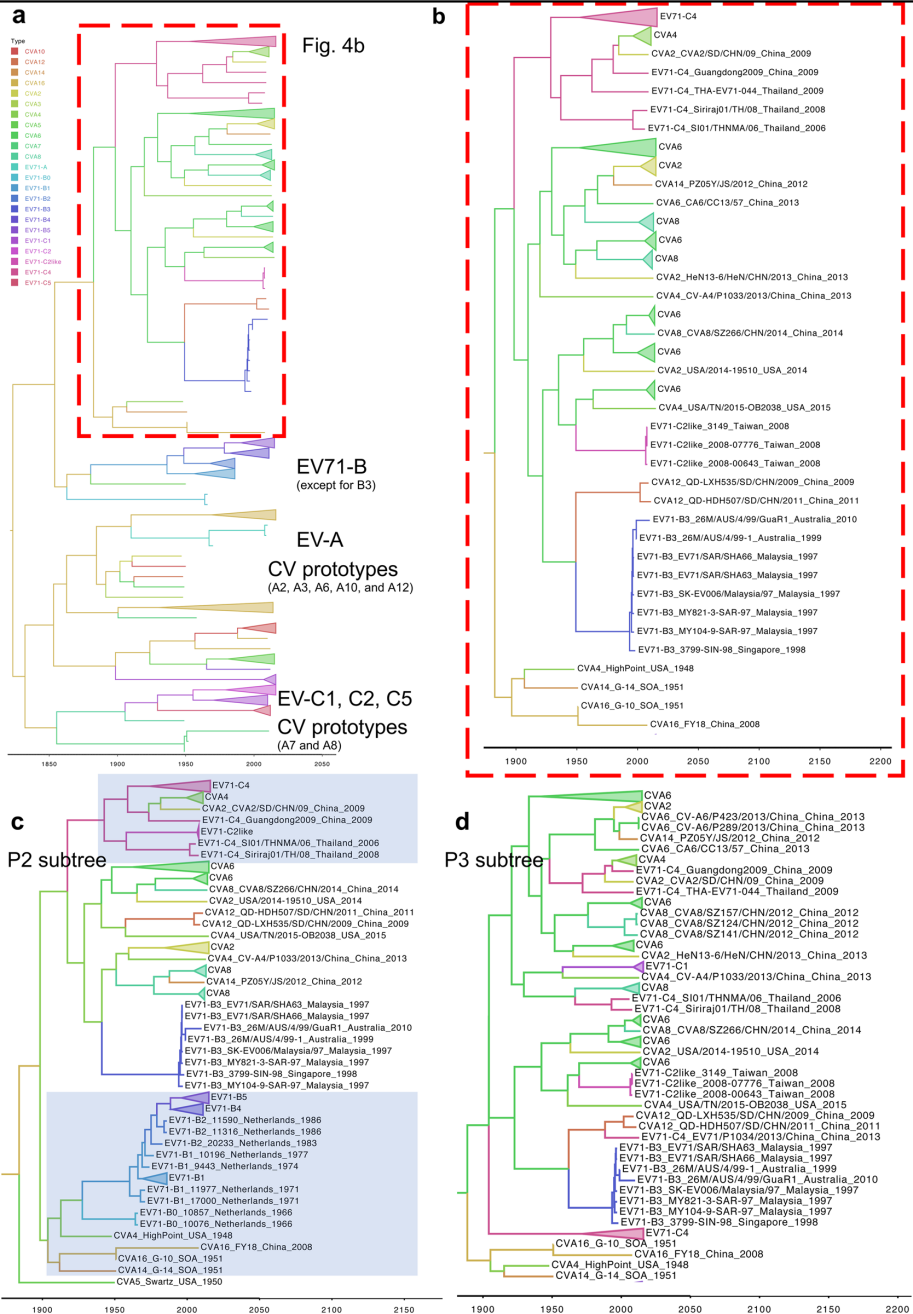
Bayesian phylogenetic tree of currently circulating EV-A viruses. Bayesian phylogenetic trees based on (**a**) and **b**. P2/P3, **c** P2 and **d** P3 regions. Genotypes/subgenotypes of EV-A71 and CVs are marked in different colors. The subtrees in (**b**–**d**) contain mixed clusters, showing intra- and intertypic recombination events among EV-A71 B3/C4 and CVs. Highlights in **c** represent the reclustering of the prototype CVs with EV-A71 B and of EV-A71 C4 with the currently circulating CV-A4

Various viruses belonging to EV-A continuously cocirculate with EV-A71 in Taiwan (Fig. 1a), and many of these non-EV-A71 viruses are recombinant with unknown parents^16^. Thus, we next examined whether EV-A71 may be involved in the recombination of non-EV-A71 viruses of EV-A. To prevent sampling bias, we collected all of the historical EV-A full-genome sequences worldwide for the following analyses. Since most recombinations in other EV-A viruses also occur outside the P1 region^16^, we evaluated EV-A71 and CV sequences in the P2/P3 coding region. We utilized Bayesian evolutionary analysis to specify the spatial-temporal relationships among these sequences. When sequences spanning from P2 to the 3′ end of the viral genome were analyzed, several clusters were observed, most of which contained clades corresponding to different serotypes of EV-A and subgenotypes of EV-A71 (Fig. 4a). One cluster of particular interest contained the EV-A71 B3, C2-like, and C4 strains as well as several currently circulating viruses (Fig. 4a, red rectangle). The details of this cluster are shown in Fig. 4b. Notably, this cluster was proximal to a second cluster containing the prototype sequences of CV-A4, CV-A14, and CV-A16, indicating a potential role of these viruses as recombination parents. Because the evolutionary paths of the circulating strains in phylogenies might be biased by a series of recombination events, we emphasized the detection of incongruous genetic clusters^20^. Considering the times at which the viruses distributed in this cluster arose, EV-A71 B3 represented the oldest strain among all the branches and may represent the possible origin of the other viruses (Fig. 4b). However, more genomes (particularly of historical EV-A strains) are required to strengthen this conclusion.

**Fig. 5:**
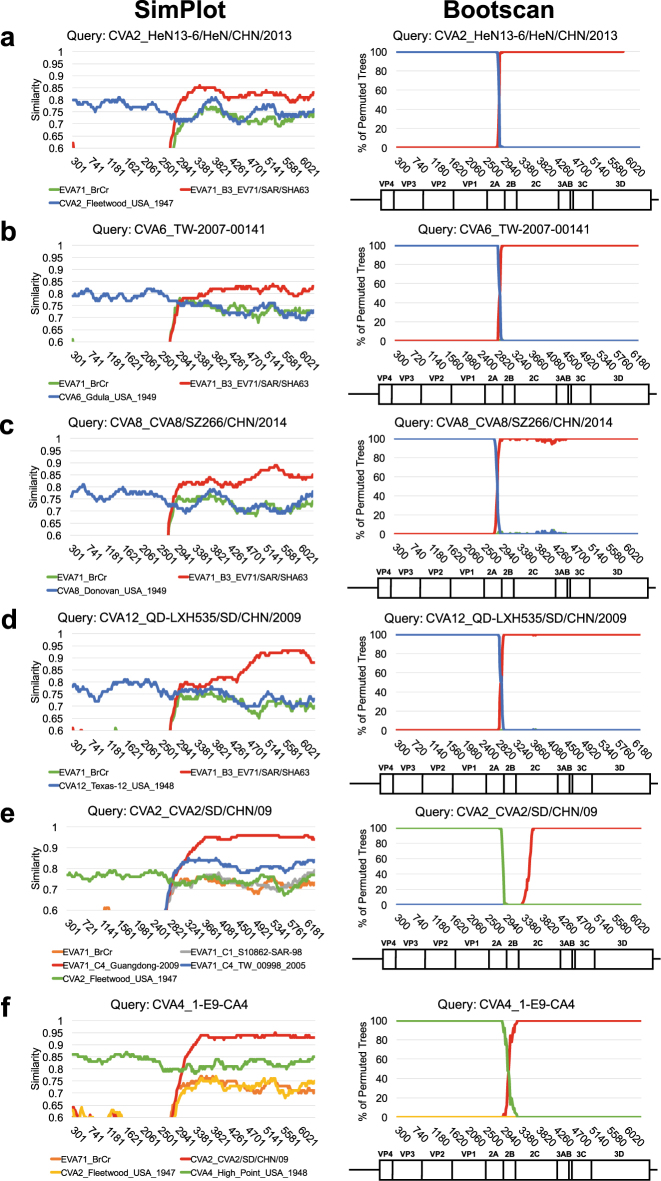
SimPlot analyses of currently circulating EV-71 and CVs. Recombination among the currently circulating CVs was examined. Using EV-A71 B3 as the reference strain, a single crossover at the P1/P2 boundary was detected by SimPlot (left panel) and Bootscan (right panel) analyses by querying (**a**) CV-A2, (**b**) CV-A6, (**c**) CV-A8, and **d** CV-A12. The roles of (**e**) EV-A71 C4 recombined with CV-A2 and **f** intertypic recombination of CV-A2 and CV-A4 are further examined. The references used in each analysis are marked in different colors, and coding nucleotide positions corresponding to the viral genome are indicated

We next compared the locations of these recombinant viruses within the phylogenies constructed using either the P2 or P3 sequence. In the P2 phylogeny, the prototype strains of CV-A4, CV-A14, and CV-A16 were redistributed to another cluster consisting of most strains of genotype B (Fig. 4c, lower), indicating the high sequence similarity among these viruses, which may have promoted the emergence of the EV-A71 B3 strain through intertypic recombination. In the EV-A71 B3-containing cluster, currently circulating rather than prototype strains of CV-A2, CV-A6, CV-A8, and CV-A12 were found. The majority of currently circulating CV-A4 was distributed to another EV-A71 C4-containing cluster, suggesting that additional recombination events involving EV-A71 C4 may occur (Fig. 4c, upper). In contrast, all viruses mixed together without clear assortment in the P3 phylogeny (Fig. 4d). This sporadic distribution indicated that most viruses may have similar P3 regions. Thus, recombination may occur among the cocirculating viruses in the P2 region, which could result in a common P3 region shared by these viruses. To verify this hypothesis, SimPlot analyses were carried out to examine the recombination of currently circulating CVs (Fig. 5). When EV-A71 B3 was incorporated as the reference strain, a single crossover was found in the P1/P2 boundary when CV-A2, CV-A6, CV-A8, and CV-A12 were analyzed (Fig. 5a–d). The role of EV-A71 C4 in the recombinant CV-A4 and an additional intertypic recombination between CV-A4 and CV-A2 were also confirmed (Fig. 5e, f). To eliminate sampling bias, consensus sequences of these recombinant strains from EV-A71 B3, B5, C2-like, and C4, and CV-A2, CV-A4, CV-A6, CV-A8, CV-A12, and CV-A16 were further generated for comparison to the prototype strains of EV-A71 and CVs. Eleven EV-A71-like signatures were identified in the circulating strains of EV-A71 C2-like and C4 and CV-A2, CV-A4, CV-A6, and CV-A12, but not their prototype strains, except for CV-A4 and CV-A16 (Table 2). Thus, in addition to CV-A16^14,18,19,21^, CV-A4 might be another potential recombination parent of these currently circulating viruses. Consistent with the results of the Bayesian phylogenetic tree (Fig. 4d) and SimPlot predictions (Fig. 5), all signatures were located in the P3 region (Table 2), which might be caused by the intensive recombinations in the P2 region. Such signatures cannot be found in the circulating CV-A16 strain, which has been reported to be recombinant with EV-A71 genotype A^16^, or in the EV-A71 B5 strain, which evolves independently of other viruses. Both strains carry sequences similar to those of the EV-A71 prototype strain.

**Table 2:** Amino acid positions of 11 signatures carried by circulating strains of recombinant EV-A viruses

Gene	3A	3C		3D							
Position	39	36	95	44	76	94	134	138	368	428	451
**Consensus sequence of circulating strains**											
EV-A71 B3	D	V	S	H	E	Q	T	V	N	Q	Y
EV-A71 C2-like	.	.	.	.	.	.	.	.	.	.	.
EV-A71 C4	.	.	.	.	.	.	.	.	.	.	.
CVA2	.	.	.	.	.	.	.	.	.	.	.
CVA4	.	.	.	.	.	.	.	.	.	.	.
CVA6	.	.	.	.	.	.	.	.	.	.	.
CVA8	.	.	.	.	.	.	.	.	.	.	.
CVA12	.	.	.	.	.	.	.	.	.	.	.
CVA16	E	I	T	T	Q	K	V	T	T	E	F
EV-A71 B5	E	.	.	T	Q	K	V	.	T	D	F
**Prototype strains (Strain, Country, Year)**											
EV-A71 A (BrCr, USA, 1970)	E	I	T	T	Q	K	V	T	T	E	L
CVA2 (Fleetwood, USA, 1947)	E	I	T	T	Q	K	V	T	T	E	F
CVA4 (HighPoint, USA, 1948)	.	.	.	.	.	.	.	.	.	.	.
CVA6 (Gdula, USA, 1949)	E	I	T	T	Q	K	A	T	T	E	F
CVA8 (Donovan, USA, 1949)	.	.	.	T	.	K	.	.	T	E	F
CVA12 (Texas-12, USA, 1948)	E	I	T	T	Q	K	V	T	T	E	F
CVA16 (G-10, SOA, 1951)	.	.	.	.	.	.	.	.	.	.	.

Signatures identified from the EV-A71 B3 strain were found in consensus sequences of circulating strains in EV-A71 genotypes C2-like and C4, and CV serotypes A2, A4, A6, and A12

## Discussion

**Fig. 6:**
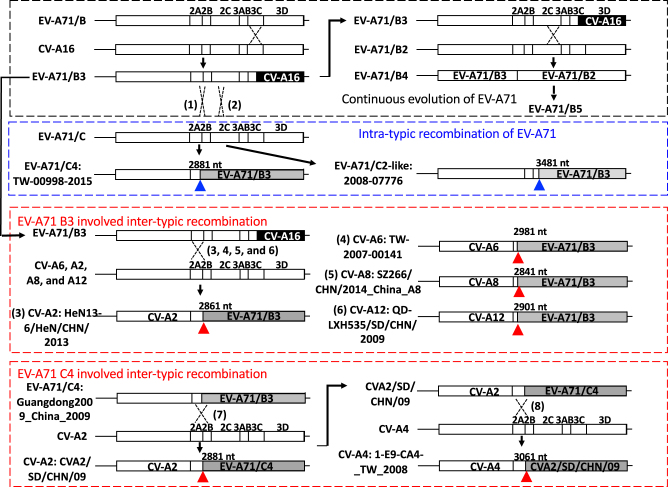
EV-A71 exhibited intensive inter- and intratypic recombination. A simplified flowchart to illustrate several recombination events identified in this study. The currently circulating EV-A71 B5 strain was derived from the B4 strain, which was an intratypic recombinant virus of EV-A71 genotype B (black rectangle). The previously active EV-A71 B3 strain that carried the CV-A16-like 3D genome also exhibited intratypic recombination (blue rectangle), resulting in the emergence of C4 (#1) and C2-like strains (#2). In addition, intertypic recombination (upper red rectangle) between EV-A71 B3 and several CVs is also shown (#3–6). EV-A71 C4-related intertypic recombination occurred in ~2009 to generate a particular CV-A2 strain (CVA2/SD/CHN/09), which further recombined with CV-A4 to generate the currently circulating CV-A4 (#7 and #8, lower red rectangle). Breakpoints corresponding to the coding nucleotide positions of each recombination event are indicated

In this study, we found that many currently circulating EV-A strains have undergone recombination and that EV-A71 B3 may have played a central role in this process, based on the latest published database of EV-A full-length genomes (Fig. 6). It is expected that more genomes will be published and will be added to this simplified flowchart. Through a series of intra- and intertypic recombinations, EV-A71-like signatures were found to be widely present in many currently circulating EV-A viruses (Table 2). Although the impact of these signatures on viral replication remains unclear, their prevalence in various EV-A viruses may have applications in the development of broad-spectrum antivirals.

### Role of recombination in the evolution of EV-A71

The classification of various EV-A71 strains was based on the nucleotide sequence diversity of the VP1 gene (genetic variation less than 12 and 19% for subgenotype and genotype, respectively). The overall identity of amino acid sequences among these viruses still reached 94%^5^. The effects of mutations and recombinations on EV-A71 evolution have been extensively discussed, and although several VP1 amino acids are under positive selection, EV-A71 may be subjected to strong negative selection, which theoretically should result in a stabilized and purified virus^7,10^. Thus, recombination may have played an important role in the appearance of diverse EV-A71 strains such as B3, B4, C2, and C4 since 1997^13,14,18,19^. Intratypic recombinations have also occurred in different EV species, with a higher frequency in EV-B species than in EV-A and EV-C^22^. Here, by analysis of full-genome sequences collected in Taiwan from 1998 to 2016, we determined when and where recombination occurred and how these events could have led to the emergence of the different strains associated with EV-A71 outbreaks. Although published genomes have limited value as a proxy for the actual viral population, several recombination events can be revealed by analyzing the phylogenetic relationships among EV-A71 subgenotypes. We did not rule out the importance of mutation during EV-A71 evolution^23^. Instead, a combination of both recombination and mutation may result in the rapid switching between different EV-A71 strains within a short time period. In the future, more genomes will be needed to decipher the evolutionary history of EV-A71.

### Extensive recombination in cocirculating viruses

Sequences with high similarity to those of the prototype strains CV-A8 and CV-A16 were found in the EV-A71 C2 and B3/C4 strains, respectively^13,14,18,19^. However, considering the requirement of coinfection for recombination, it is possible to obtain the “non-self” genome from cocirculating viruses^15^. In addition, as one of the countries having cocirculation of multiple EV-A71 subgenotypes and several EV-A viruses, Taiwan represents a good niche for clarifying the relationships among cocirculating viruses^8^. In this study, we proposed that the emergence of the EV-A71 C2-like and C4 strains may be explained by intratypic recombination with the B3 strain. Although the presence of the B3 strain in Taiwan has not been previously documented, it was involved in the recombination of the B4 strain, which caused severe outbreaks in the early 2000s^12,13^. The intensive recombinations were not EV-A71-specific and could also be found in several currently circulating EV-A viruses through intertypic recombinations. Among these viruses, only CV-A2, CV-A4, and CV-A6 have been documented in Taiwan. However, all these viruses have been reported to be common causes of HFMD in China and are recombinogenic with EV-A71^24^. Interestingly, a novel EV-A71 genotype C strain with a mosaic genome structure has been identified in Germany and Denmark^25,26^. This new strain had a C1-like VP1 region; however, the 5′ untranslated region and the P2/P3 region showed higher similarity to EV-A71 B3/C2-like and C4, respectively. This new EV-A71 strain may have been generated by recombination of the locally circulating C1 strain with the imported C4 strain that became dominant recently^27^. Although intensive recombination of cocirculating EV-A viruses with EV-A71 has been observed, there are some exceptions. Both CV-A5 and CV-A10 are commonly detected by the Taiwan enterovirus surveillance system; however, no recombination with EV-A71 has been observed. Instead, a close relationship in the nonstructural region, possibly caused by intertypic recombination between circulating CV-A5 and CV-A10, has been reported^28^. Therefore, recombination between cocirculating viruses may be more common than expected, and full-genome sequencing rather than sequencing of VP1 only should be considered when encountering a new epidemic.

### Hot spots for recombination: functional impact of genome reassembly

In all recombination events identified in this study, breakpoints were mapped to the region extending from the P1/P2 boundary to the 2C region (Fig. 6a), suggesting the existence of recombination hot spots. Thus, recombination can result in genome reassembly of the structural and nonstructural regions. The restricted location of the breakpoints may have resulted from natural selection. Delicate cooperation among picornavirus viral proteins and genomes is required for productive viral replication^29^. Because the genome of EV-A can be directly translated into a polyprotein and then undergo proteolytic cleavage, changes in the functional entities by recombination could be deleterious to the virus. For example, viral 3D^pol^ is required for viral replication and recognizes several *cis*-elements throughout the genome^29^. In the case of EV-A71 B3, due to the presence of the CV-A16-like 3D^pol^, the virulence was decreased when compared with those of EV-A71 B4 and CV-A16 in mouse model infections^21^. Therefore, the EV-A71-like signatures that consist of EV-A71 genotype B-like P2 and CV-A16-like P3 regions may have been less favored and discarded in the evolution of EV-A71 genotype B (Fig. 6a, black rectangle). However, as we have shown here, EV-A71-like signatures are tolerated by EV-A71 genotype C and several CVs of EV-A, and the numbers of appropriate recombination acceptors could keep increasing^25,26^. Because a high sequence similarity is preferred for copy-choice recombination, as detailed in the widely accepted model of RNA virus recombination^15^, the sequence identity between the EV-A71 C4 and B5 strains should definitely be higher than that between the EV-A71 and EV-A viruses. Restricted recombination under natural selection may explain why EV-A71 C4 and B5 cocirculated but did not recombine, exhibiting independent evolution. We currently have no evidence to conclude whether recombination may be beneficial for the virus; however, the ratio of recombinant CV-A6-associated HFMD has increased worldwide^24,30^. Here, we provide only evidence demonstrating the shared nonstructural proteins of cocirculating EV-A viruses. Through the establishment of sequence databases that integrate complete sets of full-genome sequences, we might able to predict what kinds of genome assembly and possible recombinants might appear in the future.

## Materials and methods

### Specimen collection and sequencing of EV-A71

All of the 64 EV-A71 clinical specimens isolated from 2005 to 2016 were provided by the Linkou Chang Gung Memorial Hospital, Taiwan. Regardless of the illness diagnosed, we randomly picked clinical samples from epidemics in this time span. To prevent contamination, amplified viral stocks from human rhabdomyosarcoma cells were used for full-genome sequencing. Viral genomes were recovered using TRIzol LS reagent (Thermo Fisher Scientific, Waltham, MA, USA) according to the manufacturer’s instructions. The 59 samples collected before 2014 were sequenced by Sanger sequencing. Oligo-(dT)_20_ was used to prepare poly(A)-containing viral cDNA using a ReverTra Ace -α- kit (Toyobo, Osaka, Japan). Overlapping amplicons covering the entire viral genome were amplified by different sets of primers^18,31^, and genome assembly was carried out using SeqMan software (DNASTAR, Inc., Madison, WI, USA). The five samples collected after 2014 were sequenced using the Illumina HiSeq platform for next-generation sequencing (NGS). NGS data preprocessing included the removal of low-quality and host reads. Using the Taiwanese B5 and C4 strains as an initial template, the viral genomes were assembled by an iterative mapping approach^32^. A total of 64 genomes obtained in this study were deposited in GenBank with accession numbers MG756691–MG756754.

### Data collection for EV-A genomes from ViPR and recombination analysis

Eight hundred thirty-one EV-A71 genomes worldwide were initially retrieved from ViPR in September 2017. Sequences with ambiguous nucleotides or without known sampling dates and countries were removed. To reduce redundancy, we randomly selected 10 sequences with the same genotype and isolation year from each country. We then collected 427 EV-A71 sequences including all Taiwanese strains for analysis (Supplementary Table 1). Moreover, 780 complete CV genomes (belonging to EV-A) were downloaded from ViPR. After data preprocessing, 351 CV genomes were collected. Details of the analyzed sequences are shown in Supplementary Table 2. Recombination between the EV-A71 and CV genomes in this study was detected using SimPlot (version 3.5.1) with a sliding window size of 600 nt and a step size of 20 nt^33^. To identify genomic signatures associated with detected recombination in this study, a consensus sequence for each of the serotypes/genotypes was generated by using the Cons tool with the default setting from EMBOSS^31^.

### Phylogenetic tree analysis

The ML method based on the Hasegawa–Kishino–Yano (HKY) model was performed to infer the evolutionary history^34^. The percentage of replicate trees in which the associated taxa clustered together in a bootstrap test with 1000 replicates was calculated. All positions with less than 95% site coverage were eliminated. Evolutionary analyses were conducted in MEGA7^35^. Furthermore, the Bayesian phylogenetic tree was inferred by BEAST^36^ with BEAGLE^37^ to estimate the maximum clade credibility (MCC) tree under the HKY model. Based on our collected sequences, we generated 50 million Markov chain Monte Carlo (MCMC) chains with 10% burn-in. One MCC tree was constructed for every 25,000 chains, and a single consensus tree was summarized from these MCC trees. MCMC was also used in BEAST to estimate the time of divergence for each alignment.

## Electronic supplementary material

Supplementary Table 1

Supplementary Table 2
